# Retroperitoneal Fibrosis: A Puzzle of Elusive Causal Link

**DOI:** 10.7759/cureus.56220

**Published:** 2024-03-15

**Authors:** Anna Paola Gagliardi, Sara Rotunno, Daniele Romanello

**Affiliations:** 1 Internal Medicine, University of Rome "Campus Biomedico", Rome, ITA; 2 Internal Medicine, Ospedale San Pietro Fatebenefratelli, Rome, ITA; 3 Internal Medicine, Ospedale San Pietro Fatebenefratelli, Roma, ITA

**Keywords:** retroperitoneal fibrosis, idiopathic retroperitoneal fibrosis, obstructive renal failure, ureteral obstruction, ureteral stents, rpf, ormond's disease

## Abstract

Retroperitoneal fibrosis (RPF) is a rare condition characterized by the presence of fibro-inflammatory tissue surrounding the abdominal aorta and iliac arteries, often leading to the involvement of adjacent organs such as the ureters and inferior vena cava. We present a case report of a 56-year-old Caucasian woman with a complex medical history, including Hodgkin's lymphoma treated with chemotherapy and radiotherapy (31 years before), a significant smoking history, and a current presentation of acute kidney injury with oliguria, edema, and hypertension. Initial diagnostic considerations included rapidly progressive glomerulonephritis, supported by clinical and imaging findings. However, the absence of specific autoantibodies and the presence of bilateral calyx-pelvic dilation raised questions regarding alternative diagnoses. Imaging studies, including CT, contrast-enhanced CT, and subsequent MRI, revealed periaortic and paracaval adipose tissue thickening suggestive of periaortitis, leading to a diagnosis of retroperitoneal fibrosis. The multifactorial etiology, including previous radiation therapy, lymphoma history, and significant smoking, posed challenges in establishing a definitive causal link. Despite extensive investigations, including laboratory tests and imaging modalities, no single etiological factor could be conclusively identified. This case underscores the diagnostic complexity of RPF, especially in the presence of multiple potential risk factors, and highlights the importance of considering this condition in the differential diagnosis of patients presenting with renal dysfunction and obstructive uropathy.

## Introduction

Retroperitoneal fibrosis (RPF) refers to a rare condition characterized by the presence of fibro-inflammatory tissue around the abdominal aorta and iliac arteries. This tissue can spread into the remaining retroperitoneal space, involving other abdominal organs such as the ureters (most frequently affected) and the inferior vena cava [1-3]. The first case of retroperitoneal fibrosis was described in the literature in 1905 by the urologist Albarran. However, it was J.K. Ormond in 1948 who first provided a detailed description of two cases and defined this new clinical entity [1]. The etiology of RPF remains largely unclear to date. Neoplasms (such as lymphomas, sarcomas, gastrointestinal and breast carcinomas), infections, radiotherapy, previous abdominal surgeries, and the use of certain medications are possible triggering causes but account for less than one-third of cases [2,3]. In the majority of cases, retroperitoneal fibrosis is classified as idiopathic retroperitoneal fibrosis (IRF). This condition can manifest either in isolation or in the context of other autoimmune diseases (such as small vessel vasculitis, rheumatoid arthritis, Hashimoto's thyroiditis); in a 2011 study conducted on 204 patients with RPF, approximately 9.8% of those examined were found to have a concurrent autoimmune disease, predominantly involving the thyroid [4,5]. New evidence suggests an association with IgG4-related disease (IgG4-RD), an immune-mediated disorder that can involve various structures (such as the pancreas, lymph nodes, and biliary tract), characterized by the infiltration of IgG4-producing plasma cells and the development of a fibro-inflammatory disorder [4,6,7]. IRF is often assimilated with perianeurysmal retroperitoneal fibrosis (PRF) and inflammatory abdominal aortic aneurysm (IAAA). All these conditions indeed fall within the spectrum of chronic periaortitis and are histologically characterized by the presence of fibrosis and chronic inflammation involving the adventitia, sparing the media and intima, which are often affected by atherosclerotic changes [4].

The clinical picture of RPF is nonspecific and can vary depending on the involvement of different organs. Nevertheless, the most common clinical presentation is obstructive nephropathy associated with varying degrees of renal insufficiency. Lower back pain is a frequent manifestation of this condition and is observed in approximately 90% of patients [2,8].

Given the clinical presentation, it is necessary to consider RPF in the differential diagnosis of other causes of obstructive nephropathy such as nephrolithiasis, ureteral stenosis, or extrinsic compression of other origins. Suspicion arises in the case of ultrasound findings of bilateral ureteral dilation in the absence of a mechanical obstruction, confirmed by CT and/or MRI. However, in some cases, a retroperitoneal tissue biopsy may be necessary [5,6,8].

The goal of therapy is to remove the obstruction by implanting stents and to control progression and potential exacerbations through immunosuppressive agents such as corticosteroids, tamoxifen, cyclophosphamide, cyclosporine, and mycophenolate mofetil. In selected cases, surgical intervention may be necessary [8].

## Case presentation

The patient was a 56-year-old Caucasian woman who presented to the emergency department with a recorded hypertensive peak associated with mild edema and oliguria for approximately 24 hours. She reported no current or previous abdominal or lower back pain, fever, dysuria, or stranguria. She was not on any home therapy.

Her medical history included Hodgkin's lymphoma diagnosed 31 years ago and treated with chemotherapy and both supra- and subdiaphragmatic radiotherapy (documentation missing).

Upon admission to the emergency department, vital signs showed a blood pressure of 180/100 mmHg, heart rate of 110 bpm, body temperature of 36°C, and oxygen saturation in ambient air of 96%. The general physical examination was unremarkable except for mild edema. Giordano's sign was bilaterally negative.

Laboratory tests revealed acute renal failure with good acid-base compensation (Creatinine 5.16 mg/dL) and leukocytosis (WBC 13.91 x 10^3^/μL) (Table 1).

**Table 1:** Patient blood analysis and urinalysis at admission. Blood sample analysis and urinalysis show acute kidney impairment and signs of inflammation.

Investigation	Patient value	Reference value
Red blood cells (RBC)	3.87 10^6^/µL	4–5
Hemoglobin (Hb)	11.3 g/dL	12–14.4
White blood cells (WBC)	13.91 10^3^/µL	4.3–10.3
Neutrophils (N)	10.04 10^3^/µL	2.1–6.5
Creatinine (Crea)	5.16 mg/dL	0.51–0.95
Azotemia	86 mg/dL	15–50
Potassium (K^+^)	4.23 mEq/L	3.5–5.1
Sodium (Na^+^)	128 mEq/L	135–148
C-reactive protein (CRP)	68.3 mg/L	0.1–5
pH	7.4	7.38–7.42
Partial pressure of arterial carbon dioxide (pCO_2_)	35 mmHg	35–45
Partial pressure of arterial oxygen (pO_2_)	84 mmHg	80–100
Bicarbonate (HCO_3_^-^)	21.7 mmol/L	21–28
Base excess (BE)	–3.1 mmol/L	–2 to +2
Urine nitrite	Absent	Absent
Urine protein	30	0–20
Urine blood	189 n°/µL	0–15
Urine leukocytes	1273 n°/µL	0–18
Urine bacteria	2716 n°/µL	0–500
Urine epithelial cells	77 n°/µL	0–20
Urine protein over 24 hours	160 mg/dL	10–150

Urinalysis showed traces of protein, the presence of red blood cells (189/μL), WBC (1273/μL), and bacteria (2716/μL). Urine culture showed no growth of common germs or fungi. Twenty-four-hour proteinuria was normal, and antinuclear antibodies (HEP II), anti-dsDNA, pANCA, cANCA, and ENA were negative.

Abdominal ultrasound revealed mild diffuse corticomedullary hyperechogenicity bilaterally associated with moderate-grade dilation of the calyx-pelvic cavities bilaterally in the absence of images attributable to lithiasic concretions >5 mm. These findings were not present on the abdominal ultrasound the patient had privately performed 6 months earlier for constipation and abdominal bloating. Chest X-ray showed mild bilateral pleural effusion.

The patient underwent bladder catheterization and received hydration, diuretic (furosemide), and antibiotic therapy (ceftriaxone 2 g) as well as antihypertensive therapy with ramipril 5 mg/day. Subsequently, due to failure to achieve normal blood pressure values, therapy with manidipine hydrochloride 20 mg/day was initiated.

In the following days, laboratory tests showed worsening renal function (creatinine 5.32-7.65 mg/dL) with almost stable neutrophilic leukocytosis and reduced C-reactive protein (white blood cell count 13.8 thousand/μL, of which neutrophils 10.82 thousand/μL, C-reactive protein 30.5 mg/L).

**Figure 1:**
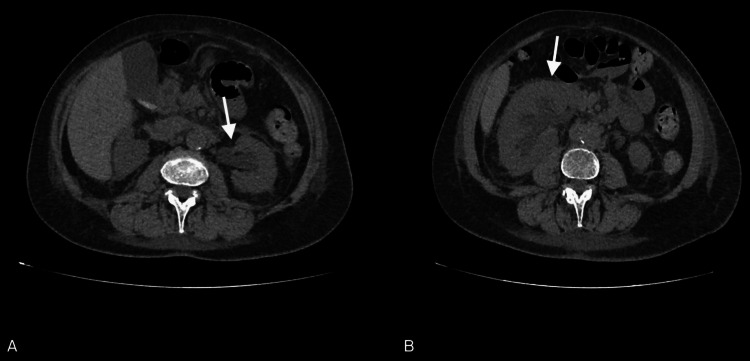
CT abdomen: left caliceal, pelvic, and ureteral dilatation (arrow in A) and right kidney medialized and swollen (arrow in B). There is modest dilatation of the calyceal cavities and the renal pelvis, as well as of the proximal tract of the ureter in the absence of definite images suggestive of lithiasis. Minimal thickening of the perirenal fascia is also associated (A). Diffuse heterogeneity of mesenteric adipose tissue, right kidney slightly medialized, increased in size with a swollen appearance, modest dilatation of the calyceal cavities and the right renal pelvis as well as of the proximal tract of the ureter. Thickening of the perirenal fascia is associated with heterogeneity of the anterior and posterior pararenal spaces (B).

Abdominal CT scan revealed diffuse mesenteric adipose tissue heterogeneity, medialization of the right kidney with increased size and swelling, and moderate dilation of the calyceal cavities, pelvis, and proximal segments of the ureters. Additionally, it detected thickening of the perirenal fascia with heterogeneity of the anterior and posterior pararenal spaces and thickening of adipose tissue in the para-aortic and paracaval regions (Figure 1).

**Figure 2:**
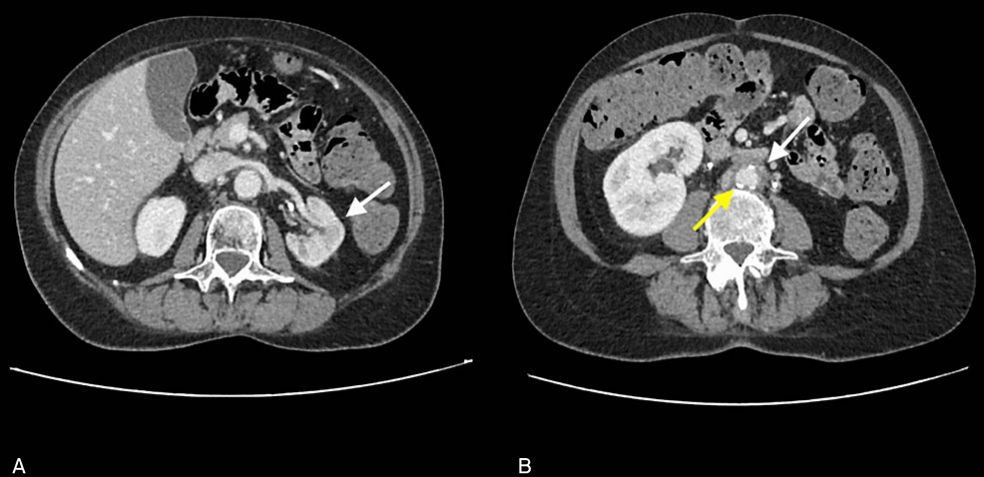
CT abdomen with contrast enhancement: resolution of obstructive nephropathy (arrow in A), thickening of periaortic and pericaval adipose tissue (white arrow in B), aortic atherosclerosis (yellow arrow in B). Bilateral pyelovesical stents in place; no dilatation of the calyceal-pelvic cavities observed. The left kidney is of reduced size with thinned parenchymal thickness (A). The right kidney is enlarged, albeit to a lesser extent compared to the previous reference. Thickening of adipose tissue in the para-aortic and paracaval regions is observed (B).

Subsequently, the patient underwent bilateral JJ ureteral stent placement and was started on corticosteroid therapy with 60 mg of methylprednisolone. Laboratory tests performed the day after the procedure showed a rapid decrease in creatinine levels (1.38 mg/dL; BUN 61 mg/dL). In consideration of the progressive improvement in renal function, it was possible to perform a contrast-enhanced abdominal CT scan to define the causes of bilateral ureteral obstruction (Figure 2). The exam showed resolution of the obstruction and thickening of adipose tissue in the para-aortic and paracaval regions.

**Figure 3:**
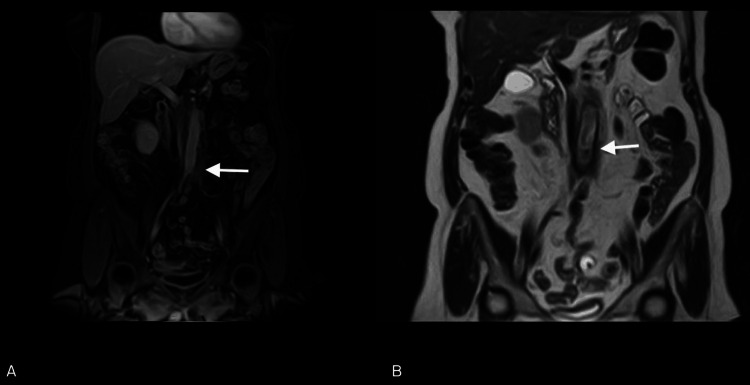
MRI abdomen with contrast enhancement: periaortic thickness (arrow in A) with sign of inflammation/activity (arrow in B). Thickening of the adipose tissue in the peri-aortic area, forming a "cuff," with a maximum sub-centimeter thickness, extending along the sub-renal abdominal aorta to the carrefour, involving the root of the left common iliac artery. Signal characteristics are low on T1-weighted images (A) and intermediate to high on T2-weighted images (B), consistent with inflammatory/active tissue characteristics. Millimetric lymph node formations are present in the common iliac area, showing restricted diffusivity.

For a better characterization of the thickening of the para-aortic adipose tissue, an MRI was performed, confirming the thickening of the peri-aortic adipose tissue, sleeve-like, extending along the subrenal abdominal aorta to the common iliac artery root on the left. This thickening showed intermediate/high signal intensity on T2 and low signal intensity on T1, suggestive of inflammatory/active characteristics (Figure 3).

The patient was discharged with instructions to undergo ureterolysis surgery and a targeted biopsy of the periaortic fibrotic tissue. Following the procedure, the ureteral stents were removed, and the patient was referred to a specialist for specific therapy. The biopsy results ruled out a neoplastic nature and confirmed the diagnosis of RPF: "Fibrous tissue with areas of sclerosis and foci of chronic inflammation. Adipose tissue free from significant histological alterations."

## Discussion

The clinical presentation with oliguria, edema, arterial hypertension, microhematuria, proteinuria, and progressive renal failure initially directed our diagnosis toward nephritic syndrome due to rapidly progressive glomerulonephritis (RPGN); the echogenicity of the renal cortex on ultrasound supported this diagnosis. However, glomerulonephritis did not explain the bilateral calyx-pelvic dilation. The negativity of the tested autoimmune panel (antinuclear antibodies HEP II, anti-dsDNA, pANCA, cANCA, and ENA) definitively discouraged our initial diagnostic hypothesis.

In determining the obstructive nature of renal failure, imaging was crucial. A non-contrast CT scan was performed, which, confirming the calyx-pelvic ectasia already seen on ultrasound, showed bilateral proximal ureteral dilation in the absence of lithiasic concretions and revealed medialization of the right kidney. Thanks to the subsequent placement of stents and the consequent rapid recovery of renal function, a contrast-enhanced CT scan was possible: in this case, it showed para-aortic and paracaval adipose tissue thickening.

The diagnostic suspicion of disease relapse was certainly supported by the patient's history of Hodgkin's lymphoma, her previous heavy smoking habit, and previous supra- and subdiaphragmatic radiotherapy; however, there were no fever, weight loss, itching, or history of night sweats in her history.

For a better characterization of the para-aortic and paracaval thickening, an MRI was necessary, which showed extension along the subrenal abdominal aorta with involvement of the left common iliac artery root and an inflammatory nature. The presence of para-aortic inflammatory tissue, and therefore periaortitis, with traction and consequent ureteral obstruction suggested the diagnosis of RPF. In the case of our patient, previous radiotherapy and oncological history may have triggered a periaortic inflammatory process with subsequent fibrosis.

Brandt et al. evaluated 204 urological patients with retroperitoneal fibrosis, finding that periaortic inflammation was present in 5.9% of patients, previous chemotherapy in 3.9%, and previous radiotherapy in approximately 1.5%. The most interesting data undoubtedly relate to smoking habits, present in 75.6% of cases [5].

Retroperitoneal fibrosis is a rare condition that can be associated with a chronic inflammatory stimulus acting as a trigger for fibrosis development. Fibrosis alters the normal anatomy of the retroperitoneum, involving adjacent organs. In most cases, it manifests once the ureters are involved because urinary tract obstruction develops. The origin of the pathology is not yet fully understood, and this case aims to add clinical cases to increase knowledge to have more tools to explore the conditions that can give rise to this pathology. The patient in question presented with obstructive renal failure caused by retroperitoneal fibrosis. Further investigations showed that the cause of this fibrosis was attributable to periaortitis.

Identifying the causes of periaortitis is more challenging. Firstly, the patient underwent an ultrasound six months before admission to the emergency department for abdominal swelling and constipation, which did not document urinary tract obstruction or calyx-pelvic dilations. This raises the first unsolved question about the time required for retroperitoneal fibrosis to manifest clinically. Although the silent onset and asymptomatic progression until the involvement of retroperitoneal organs make it difficult to establish the speed of disease progression, the development of a hypertensive crisis, renal failure, and edema are indicative of significant progression of the disease in the six months before admission. The second question concerns the etiological origin. The histological examination, negative for proliferative diseases, excluded the hypothesis of disease relapse. Considering the clinical history of Hodgkin's lymphoma treated with radiotherapy, a post-irradiation origin of inflammation could be considered, which, however, does not seem congruent with the clinical evolution timelines. Radiological examinations ruled out aortic aneurysmal pathologies, but atherosclerosis was present, and blood tests ruled out autoimmune diseases.

Ultimately, despite the patient's clinical history of lymphoma, radiotherapy, and periaortitis, it is not possible to define the etiological origin of retroperitoneal fibrosis. However, it is possible to hypothesize that since each condition (neoplastic, radiation-induced, and vascular) can individually trigger the inflammatory reaction causing retroperitoneal fibrosis, in the future, it may be possible to identify a pro-inflammatory factor common to all three conditions as the true cause of fibrosis initiation.

## Conclusions

The diagnosis of retroperitoneal fibrosis poses a clinical challenge, often being achieved when organ involvement has already occurred. The difficulty in diagnosis stems from the multifactorial etiology that is not yet fully understood. Therefore, retroperitoneal fibrosis should be considered in the differential diagnosis whenever there is suspicion of one or more conditions that may underlie the etiology of this pathology.
